# Graves' Disease-Associated Dilated Cardiomyopathy Unmasked by Blunt Chest Trauma

**DOI:** 10.7759/cureus.35488

**Published:** 2023-02-26

**Authors:** Abhinav Karan, Pranitha Chekka, Thaer Musa, Khadeeja Esmail

**Affiliations:** 1 Internal Medicine, University of Florida College of Medicine – Jacksonville, Jacksonville, USA; 2 Cardiology, University of Florida College of Medicine – Jacksonville, Jacksonville, USA

**Keywords:** hyperthyroidism, thyroid, blunt chest trauma, heart failure, cardiomyopathy, grave's disease

## Abstract

Graves' disease can manifest with numerous cardiovascular complications, although very few cases result in cardiomyopathy. Our patient presented following a motor vehicle accident where she suffered blunt chest trauma. Her initial presentation was concerning for acute heart failure due to blunt cardiac injury, with diffuse pulmonary edema, hemodynamic instability, and an acutely reduced ejection fraction with global hypokinesis on transthoracic echocardiography. On further evaluation with thyroid function testing and autoimmune studies, she was found to have uncontrolled Graves' disease. She was subsequently started on methimazole for her Graves' cardiomyopathy. Following discharge, an outpatient cardiac MRI revealed no late gadolinium enhancement and complete recovery of her ejection fraction to normal values. This case serves to highlight the importance of a complete evaluation of cardiomyopathy and highlights an interesting case of a patient with blunt chest injury with a background of undetected Graves' cardiomyopathy.

## Introduction

Graves' disease is an autoimmune disorder characterized by thyroidal (hyperthyroidism, goiter), and extra-thyroidal (Graves' orbitopathy, pretibial myxedema, Graves' acropachy) manifestations. It typically can occur at any age, although the peak incidence is between 30-60 years of age and its annual incidence ranges from 14 to 50 per 100,000 persons [1]. Graves' cardiomyopathy is an extra-thyroidal manifestation of Graves' disease seen in less than 1% of all affected patients. It represents a frequently undetected and underestimated cause of heart failure with a reduced ejection fraction, particularly in younger female patients [2]. In this case, we highlight a case of decompensated heart failure and cardiogenic shock in a young, female patient initially presumed to be secondary to blunt cardiac injury. Based on our patient's echocardiographic findings, clinical history, and in-hospital vital signs, further underlying pathology was suspected and further workup subsequently revealed Graves' cardiomyopathy. We aim to highlight a unique presentation of previously undiscovered Graves' cardiomyopathy in the background of confounding blunt chest injury.

## Case presentation

A 37-year-old female with a history of hypertension presented to the hospital following a motor vehicular collision (MVC). She reported driving at 65 miles per hour along the highway, when she swerved into an oncoming vehicle to avoid a pedestrian, striking her anterior chest against the steering wheel. She confirmed that she never lost consciousness, however, she reported the onset of acute shortness of breath and tachypnea immediately thereafter. She exited the car and attempted to ambulate, but noted worsening dyspnea on exertion. On arrival at the emergency department, she was tachypneic and tachycardic with a respiratory rate of 32 breaths per minute and a heart rate of 124 beats per minute. Her blood pressure was 107/65. Physical examination was significant for bilateral, diffuse crackles, and an S3 heart sound, but no jugular venous distention or lower limb edema. She had no thyromegaly, or ophthalmic findings on examination. She denied orthopnea, paroxysmal nocturnal dyspnea, or lower limb edema, but noted feeling occasionally dyspneic after ambulating for longer than two to three blocks. She reported no personal or prior history of any cardiac disease.

Initial cardiac biomarkers were significant for an NT-pro BNP of 2583pg/ml (reference range: 0-125pg/ml) and a high sensitivity troponin trend of 324ng/ml, 242ng/ml, and 205ng/ml (reference range: <14ng/ml), all taken an hour apart (reference range: delta troponin <5 at 1 hour, and <7 at 3 hours). Her renal profile was unremarkable, with a creatinine of 0.51mg/dl (reference range: 0.51mg/dl - 0.96mg/dl) and normal electrolytes. She had a normal hemoglobin of 12.7g/dl (reference range: 12.0 - 16.0g/dl) with reactive leukocytosis to 122,000/cm3 (Table 1).

**Table 1:** Table demonstrating patient's initial laboratory results, cardiac biomarkers, and corresponding reference ranges.

	Patient Result	Reference Range
NT-pro BNP	2583pg/ml	0-125pg/ml
High sensitivity troponin	324ng/ml, 242ng/ml, and 205ng/ml 1 hour apart	Delta troponin <5 at 1 hour, and <7 at 3 hours
Creatinine	0.51mg/dl	0.51mg/dl - 0.96mg/dl
Hemoglobin	12.7g/d	12.0 - 16.0g/dl
White Blood Cell	122,000/cm3	40,000/cm3 - 100,000/cm3

**Figure 1:**
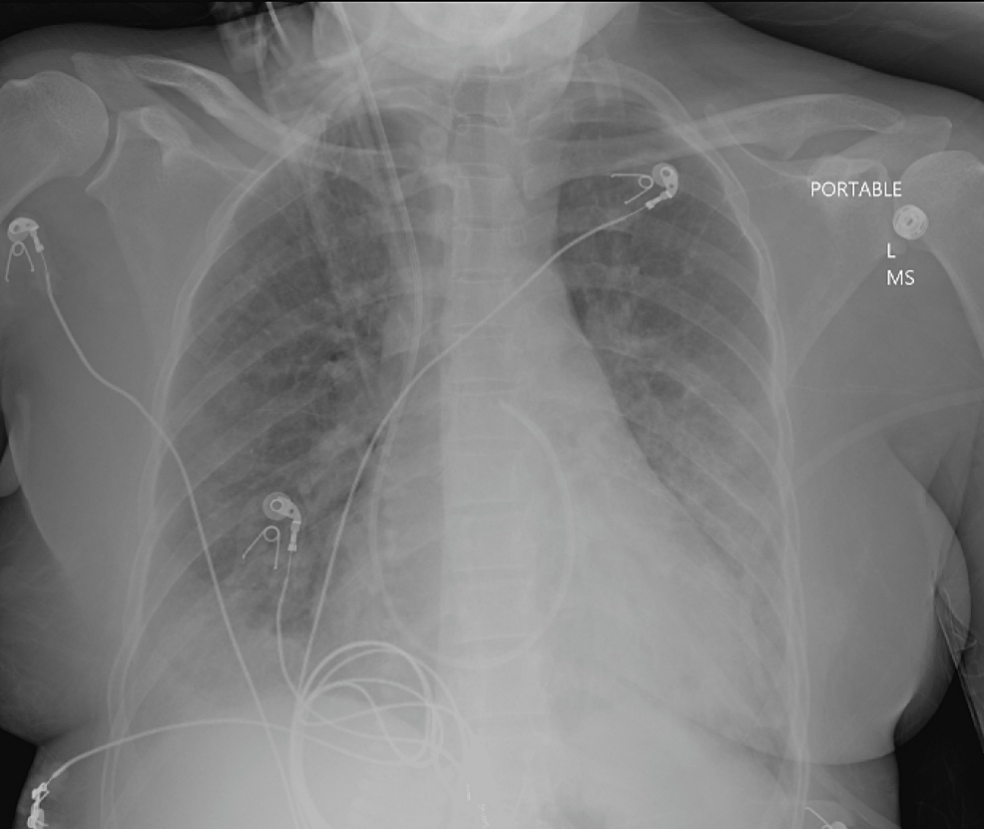
AP view of frontal chest X-ray depicting bilateral pulmonary edema and an enlarged cardiopulmonary silhouette.

A CXR revealed an enlarged cardiopulmonary silhouette, with evidence of diffuse bilateral pulmonary edema (Figure 1).

An immediate bedside transthoracic echocardiogram (TTE) revealed a reduced ejection fraction with global hypokinesis, concerning for new-onset heart failure with reduced ejection fraction. She was placed on bilevel positive pressure ventilation and was started on a nitroglycerin and furosemide infusion for the treatment of acute new-onset heart failure with a severely reduced ejection fraction.

**Figure 2:**
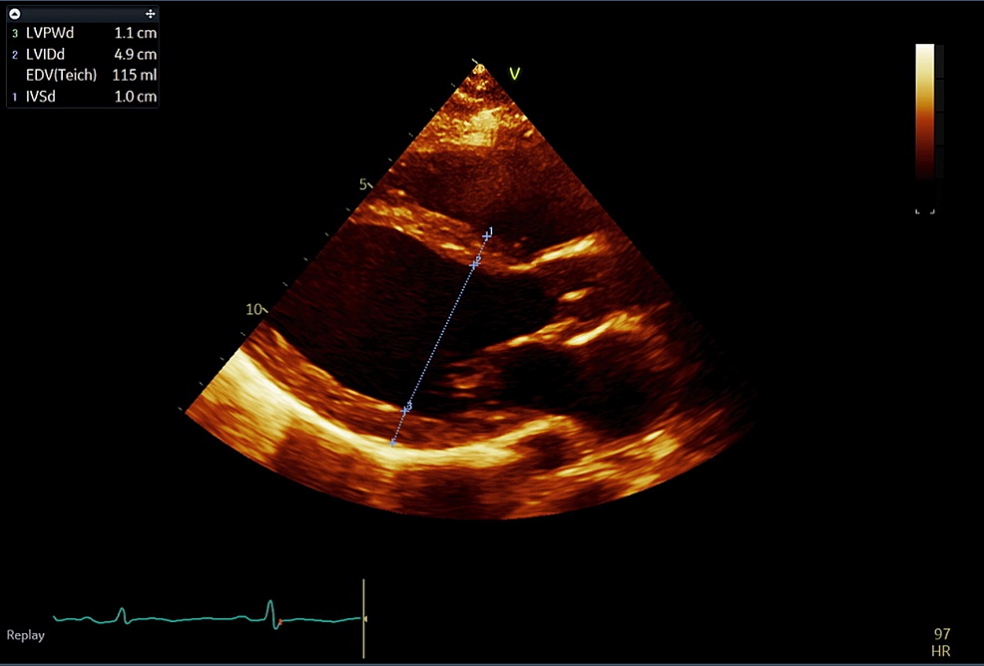
Parasternal long axis on transthoracic echocardiogram at end-diastole demonstrating mild left ventricular hypertrophy.

**Figure 3:**
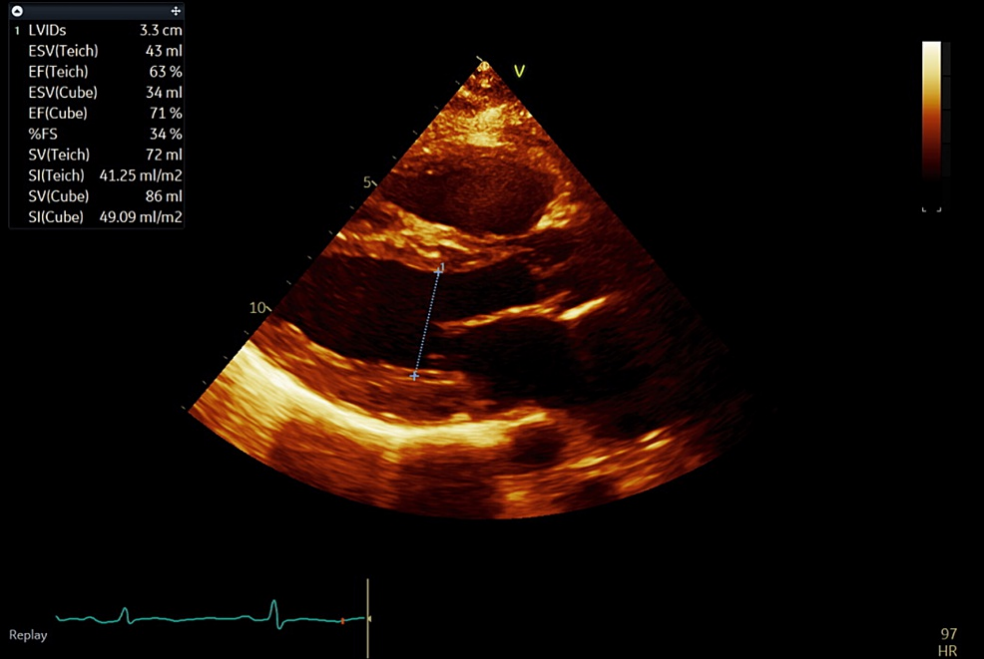
Parasternal long axis on transthoracic echocardiogram at end-systole demonstrating evidence of severely reduced ejection fraction.

**Figure 4:**
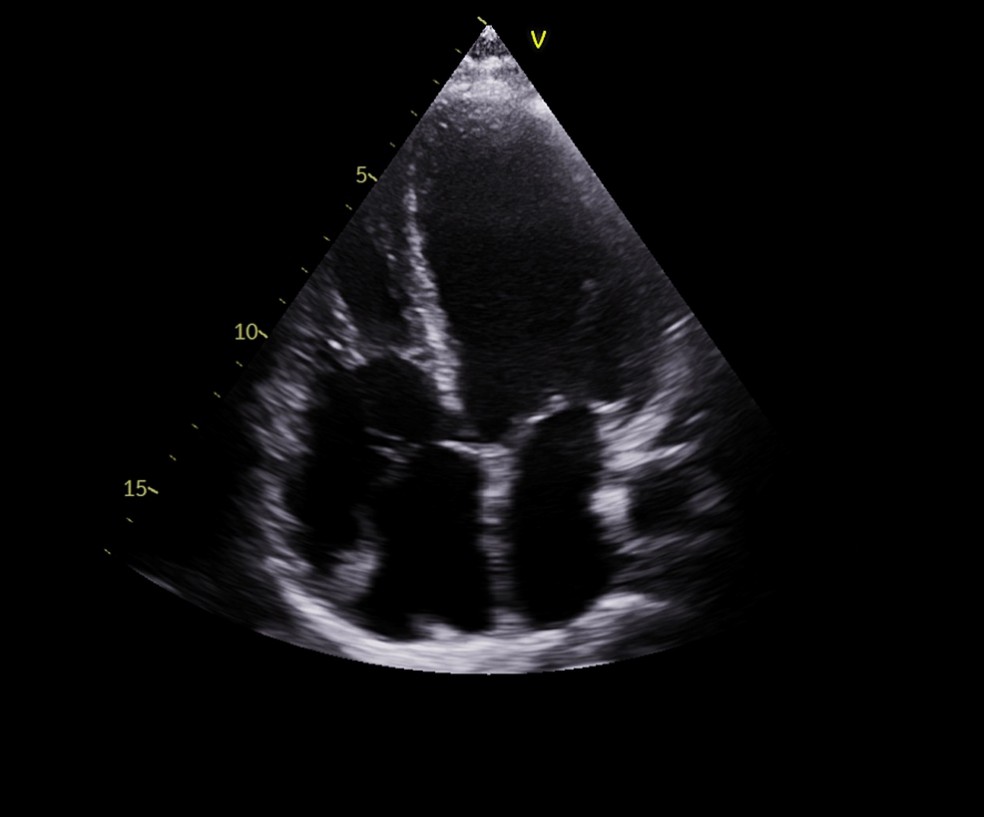
Apical four-chamber view on transthoracic echocardiogram demonstrating biatrial enlargement, global hypokinesis, and right ventricular dysfunction.

She was admitted to the cardiac care unit, and a formal transthoracic echocardiogram revealed severe biventricular and biatrial enlargement and severely reduced left ventricular ejection fraction of 25-30% (Figures 2-4 and Videos 1-2).

**Video 1:** Video of transthoracic echocardiogram in parasternal long axis view demonstrating left ventricular hypertrophy and severe left ventricular systolic dysfunction.

**Video 2:** Video of transthoracic echocardiogram in apical four-chamber view demonstrating severe left ventricular systolic dysfunction.

Also noted was extensive swirling of contrast suggestive of severe left ventricular stasis, and thickened left ventricular walls suggesting some cardiac remodeling. A right heart catheterization was pursued to assimilate data on the patient's hemodynamics and to guide diuresis. Hemodynamics revealed a right atrial pressure of 5mmHg, right ventricular systolic pressure of 49mmHg, mean pulmonary artery pressure of 34mmHg, pulmonary capillary wedge pressure of 13mmHg, pulmonary vascular resistance of 3.2 Wood Units, cardiac output of 6.5L/min, cardiac index of 3.7L/min/m^2^ and systemic vascular resistance of 873 dynes. 

**Figure 5:**
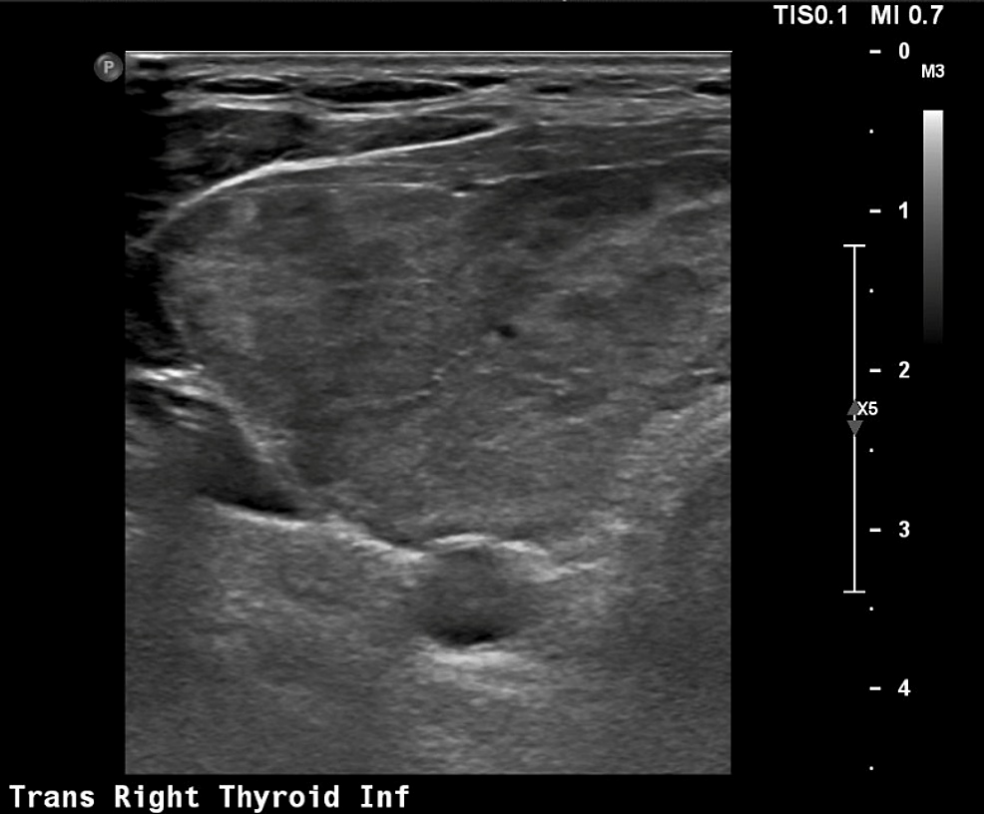
Thyroid ultrasound demonstrating significant heterogeneity and increased vascularity suggestive of thyroiditis.

On further history, the patient endorsed subjective fevers for several days prior to presentation, and her temperature on admission was 38.0^o^C. A respiratory viral panel, blood cultures, and urinalysis were unremarkable. She remained persistently in sinus tachycardia to 120-130 beats per minute. Given this hyperthermia, tachycardia, and new onset heart failure, a thyroid profile was tested. Her thyroid stimulating hormone was 0.010MIU/L (reference range: 0.270MIU/L - 4.2MIU/L), with a free T4 of 5.14ng/dl (reference range: 0.80ng/dl - 1.7ng/dl), and free T3 of 22.1pg/ml (reference range: 2.0pg/ml - 4.4pg/ml). Thyroid ultrasonography revealed diffuse enlargement of the thyroid gland with marked heterogeneity and increased vascularity, concerning for underlying thyroiditis (Figure 5).

Both thyroid peroxidase antibodies and thyroid-stimulating immunoglobulin were significantly elevated at >600 IU/ml (reference range: 0-34 IU/ml) and 1.86 (reference range: 0.0 IU/L - 0.55 IU/L) respectively (Table 2).

**Table 2:** Table demonstrating the patient's thyroid function tests and thyroid antibody titers.

	Patient Result	Reference Range
Thyroid Stimulating Hormone	0.010MIU/L	0.270 - 4.20MIU/L
Free T4	5.14ng/dl	0.80 - 1.7ng/dl
Free T3	22.1pg/ml	2.0 - 4.4pg/ml
Thyroid Peroxidase Ab	>600IU/ml	0-34IU/ml
Thyroid Stimulating Immunoglobulin	1.86IU/L	0.0-0.55IU/L

A diagnosis of Graves' disease was made and the patient was started on 25mg of daily metoprolol succinate and 10mg of methimazole three times daily. The patient demonstrated improvement in her medical condition and was subsequently discharged to outpatient follow-up on the resolution of symptoms and return to euvolemia. She remained compliant with her angiotensin-converting enzyme inhibitor (ACE-i), beta blocker, and methimazole. An outpatient cardiac MRI three months following discharge revealed no evidence of any late gadolinium enhancement to suggest evidence of ischemic cardiac injury or myocarditis and an improved ejection fraction of 55%-60%. Her thyroid function improved with a TSH of 3.12MIU/L and free T4 of 1.1ng/dl. She has thus far remained asymptomatic on medical therapy and is doing well on follow-up six months following discharge. 

## Discussion

Graves' thyrotoxicosis can manifest with numerous profound cardiovascular complications in 6% of all patients, however, less than 1% of patients present with overt heart failure [2]. In our case, the diagnosis of Graves’ disease was complicated by the confounding suspicion of blunt cardiac injury. Blunt cardiac injury is very rare, occurring in as low as 3% of all cases of blunt chest injury [3]. Graves' cardiomyopathy is essential to diagnose due to its completely reversible nature, and excellent prognosis [4]. 

Echocardiography can play a useful role in the differentiation of these conditions. In the setting of myocardial contusion, echocardiography can demonstrate dilatation of biventricular cavities, alongside regional wall motion abnormalities and features of Takotsubo’s cardiomyopathy. While our patient indeed demonstrated such findings on her echocardiogram, the thickened ventricular walls suggest cardiac remodeling which favors a chronic, underlying process. Thyrotoxicosis is suggested as the underlying etiology of this based on her thyroid profile suggests a profoundly hyperthyroid state and an autoimmune profile suggesting underlying Graves’ disease. Furthermore, underlying thyrotoxicosis is also suggested in our patient’s hemodynamic profile. It is well established that the systemic vascular resistance in hyperthyroidism is low due to beta receptor-mediated vasodilation of vasculature, as seen in our case [5]. Furthermore, underlying pulmonary arterial hypertension is evidenced by her pulmonary vascular resistance being greater than 3.0 Wood units. Interestingly, approximately 65% of patients with Graves' disease have been shown to have pulmonary hypertension [6]. The mechanism of pulmonary hypertension in Graves' disease is believed to be related to enhanced catecholamine sensitivity, causing pulmonary vasoconstriction, and subsequent increase in vascular resistance. There is also increased metabolism of pulmonary vasodilators (prostacyclins, nitric oxide) and direct autoimmune-mediated endothelial damage to the pulmonary vasculature. In the vast majority of cases, Graves' disease-mediated pulmonary hypertension is reversible with therapy to a euthyroid state [7].

This case highlights the importance of a thorough evaluation of all causes of LV systolic dysfunction even when a diagnosis seems concluded. While blunt cardiac injury is certainly a cause of LV systolic dysfunction, chronic left ventricular changes seen on bedside echocardiography suggested an underlying chronic pathophysiology. While it is prudent to mention that blunt cardiac injury can result in LV systolic dysfunction, our findings of Graves' cardiomyopathy resulted in an excellent prognosis with complete recovery of her ejection fraction.

## Conclusions

Graves' disease can present with a number of extra-thyroidal manifestations that are often difficult to diagnose. Graves' cardiomyopathy is a very rare manifestation that frequently is not considered in the investigation of acute heart failure. While our patient’s initial presentation was assumed to be secondary to blunt cardiac injury, further investigation unmasked underlying Graves' cardiomyopathy which completely recovered with anti-thyroid therapeutic strategies. Physicians must remain aware of this entity and its appropriate management as accurate diagnosis and management can result in an excellent prognosis, as highlighted by our case. 
